# Selective Reduction of Cr(VI) in Chromium, Copper and Arsenic (CCA) Mixed Waste Streams Using UV/TiO_2_ Photocatalysis

**DOI:** 10.3390/molecules20022622

**Published:** 2015-02-03

**Authors:** Shan Zheng, Wenjun Jiang, Mamun Rashid, Yong Cai, Dionysios D. Dionysiou, Kevin E. O’Shea

**Affiliations:** 1 Department of Chemistry and Biochemistry, Florida International University, Miami, FL 33199, USA; E-Mails: zhengshan@mail.sic.ac.cn (S.Z.); wjian001@fiu.edu (W.J.); mrash008@fiu.edu (M.R.); cai@fiu.edu (Y.C.); 2 Environmental Engineering and Science Program, University of Cincinnati, Cincinnati, OH 45221-0012, USA; E-Mail: dionysios.d.dionysiou@uc.edu

**Keywords:** TiO_2_ photocatalysis, CCA, chromium(VI), remediation, reduction

## Abstract

The highly toxic Cr(VI) is a critical component in the Chromated Copper Arsenate (CCA) formulations extensively employed as wood preservatives. Remediation of CCA mixed waste and discarded treated wood products is a significant challenge. We demonstrate that UV/TiO_2_ photocatalysis effectively reduces Cr(VI) to less toxic Cr(III) in the presence of arsenate, As(V), and copper, Cu(II). The rapid conversion of Cr(VI) to Cr(III) during UV/TiO_2_ photocatalysis occurs over a range of concentrations, solution pH and at different Cr:As:Cu ratios. The reduction follows pseudo-first order kinetics and increases with decreasing solution pH. Saturation of the reaction solution with argon during UV/TiO_2_ photocatalysis had no significant effect on the Cr(VI) reduction demonstrating the reduction of Cr(VI) is independent of dissolved oxygen. Reduction of Cu(II) and As(V) does not occur under the photocatalytic conditions employed herein and the presence of these two in the tertiary mixtures had a minimal effect on Cr(VI) reduction. The Cr(VI) reduction was however, significantly enhanced by the addition of formic acid, which can act as a hole scavenger and enhance the reduction processes initiated by the conduction band electron. Our results demonstrate UV/TiO_2_ photocatalysis effectively reduces Cr(VI) in mixed waste streams under a variety of conditions.

## 1. Introduction

The practice of using chemicals to protect wood products from biological degradation and deterioration has been effectively used for nearly a century. One of the most widely used wood preservatives is Chromated Copper Arsenate (CCA), a mixture of oxyanions of chromium, copper and arsenic [1]. A number of different proportions of Cr:As:Cu have been employed as wood preservatives. The role of copper in CCA treated wood is to protect the wood from the attack of bacteria and fungi; arsenic plays the role of insecticide while chromium acts as a binder of arsenic and copper to the wood surface [2]. Human exposure to these toxic metals can occur from direct contact with the wood (dermal sorption), inhalation of wood dust [3] or from CCA leachate or waste streams leading to contaminated water and soil [4]. These metals/metalloids pose significant threats to human health and the environment. The highly toxic and carcinogenic nature of arsenic is well documented [5]. An estimated 50 to 100 million people suffer or are threatened from the negative health effects caused by the ingestion of arsenic contaminated water. In aqueous media, arsenic typically exists as arsenate, As(V) and arsenite, As(III).While both As(III) and As(V) are stable and toxic, As(III) is more poisonous and more mobile than As(V).Chromium, in the third oxidation state, Cr(III), is an essential micronutrient for human health however, it is highly toxic and carcinogen in the Cr(VI) state [6]. Although less pronounced, copper can also exert adverse effects on human health at high levels of exposure despite its important function as a nutrient at trace level concentrations [7]. The co-existence of these metals can be more toxic compared to the toxicity associated with each metal individually [8].

In response to the concern over the leaching of CCA from the treated wood products, such as playground equipment, patios, picnic tables, residential uses of CCA woods were banned in USA and Canada in 2004. However, the use of CCA treated wood is still allowed for a variety of commercial, industrial and agricultural purposes. Approximately, 300 million cubic meters of wood are treated with CCA, annually, consuming nearly 500,000 tons of CCA preservative formulations [9]. The extensive and sustained use of CCA preserved wood has led to the generation of significant quantities of CCA treated woodwaste which is predicted to reach approximately 9 million m^3^/year by 2015 in USA and 2.5 million m^3^/year by 2025 in Canada [10]. Various extraction methods including supercritical fluid extraction [11], hydrogen peroxide extraction [12], EDTA extraction [13], oxalic acid extraction [14], have been reported in the literature for the separation of Cr, Cu and As from CCA treated woodwaste. Unfortunately, these methods can suffer from limitations of low extraction efficiencies, long treatment times, interference from other metals, and high cost [11,12,13,14,15]. Proper handling and disposal of CCA woodwaste is a serious challenge as ultimately these toxic metals can leach from wood through aging and weathering processes leading to discharge of the toxic metals into the environment. Landfill is still the most common fate of CCA treated woodwaste in many countries including USA and Canada as CCA wood products are not currently categorized as hazardous waste [16].Once deposited into the landfills, CCA treated products leach toxic arsenic and chromium species into the environment through aqueous runoffs and pose a serious threat to human life and to the surrounding environment [17]. Thus there is an urgent need to identify an environmentally sound and cost effective method to treat CCA contaminated leachates and explore possible strategies to recover the metals for future use.

The toxicity of these metals can be dramatically influenced by their oxidation state. In CCA, Cu and As exist as oxides of Cu(II) and As(V) [18]. Cr is present as Cr(VI) in CCA before fixation, which involves a reductive process leading to partial conversion to Cr(III) during binding with wood lignin [19]. Cr(VI) is toxic and mobile in the environment while Cr(III) is less toxic and a number of reductive processes including photocatalysis can effectively transform Cr(VI) to Cr(III) [20].Chromium species can be removed and recovered from aqueous media employing adsorption or precipitation processes [21,22]. While semiconductor mediated photocatalysis can be used to oxidize an extensive number of pollutant and toxins, a relatively limited number of examples have appeared on effective applications for reductive transformations of pollutants and toxins [23]. Among semiconductor photocatalysts, TiO_2_ is most extensively studied for water purification. Upon photo-excitation of TiO_2_ with wavelengths ≤385 nm, an electron/hole pair is produced, Equation (1), the hole can initiate oxidation through electron transfer with water, Equation (2) or a metal at the surface Equation (3), while the electron can act as a reducing agent to molecular oxygen, Equation (4) or metal ions, Equation (5):

(1) $$TiO_{2}\;\underset{hv}{⇌}\;h^{+}+\;e^{-}$$

(2) $$h^{+}+\;H_{2}O\;\to \;HO^{•}$$

(3) $$HO^{•}+\;m^{n+}\;\to \;m^{n+2}$$

(4) $$e^{-}+\;O_{2}\;\to \;O_{2}^{•-}$$

(5) $$e^{-}+\;m^{n+}\;\to \;m^{n-1}$$

A number of reports have appeared in the literature on the TiO_2_ adsorption and photocatalytic reduction of Cr(VI) [24,25,26,27,28,29,30,31,32]. TiO_2_ materials can have strong metal ion adsorption properties and are thus attractive for adsorption and photo-initiated treatment as simultaneous processes for the removal of heavy metals from the contaminated waste streams and leachates. Since adsorption of Cr(VI) at the surface of the semiconductor is critical for effective reduction during photocatalysis, it is important to examine the effects of As and Cu in the treatment strategies for CCA waste streams. In this study, we have demonstrated the effective reduction of Cr(VI) to Cr(III) using TiO_2_ photocatalyst in presence of As(V) and Cu(II). We have also investigated the influence of solution pH and CCA formulations on the reduction of Cr (VI) to Cr (III).

## 2. Results and Discussion

### 2.1. The Reduction of Cr(VI)

**Figure 1:**
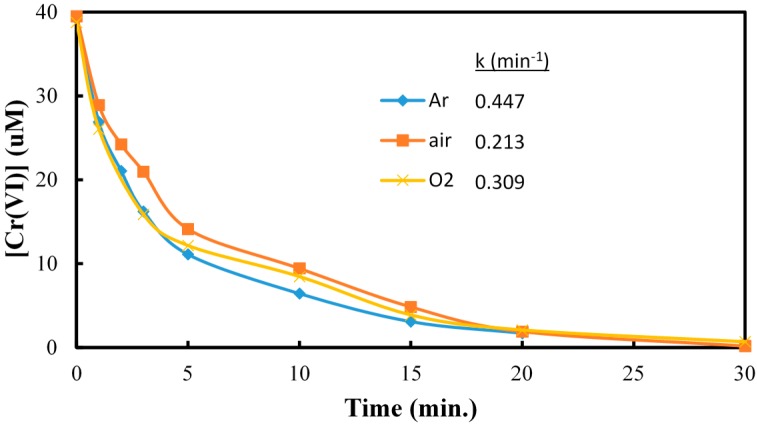
The effect of dissolved argon, oxygen and air on the reduction of Cr(VI). [Cr(VI)] = 40 µM, [TiO_2_] = 0.10 g/L, pH = 3, 350 nm.

In a typical experiment, a suspension of TiO_2_ was prepared with a CCA stock solution and transferred to a Pyrex reaction vessel. The reaction solution was purged with oxygen, air, or argon, and irradiated with UV light. Analyses were performed at specific time intervals. In the presence of TiO_2_, oxygen and UV irradiation, Cr(VI) is readily converted to Cr(III) which is measured from the difference of initial and final concentration of Cr(VI). Control experiments established the negligible reduction (<3%) of Cr(VI) in the absence of TiO_2_ and very little adsorption (<1%) of Cr(VI) on TiO_2_ in the dark. The purging of the CCA reaction solution with different gases (Ar, air, O_2_) prior and during irradiation had minimal effect on the photocatalytic reduction of Cr(VI) shown in Figure 1. These results are consistent with previous reports on the adsorption and reduction of Cr(VI) during TiO_2_ photocatalysis [33]. The pKa and speciation of arsenic and chromium metals are shown in Table 1. Copper exists as non-protonated copper oxide form.

**Table 1:** pKa and speciation of chromium and arsenic.

Arsenic (As)		Chromium (Cr)
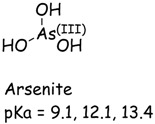	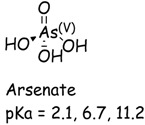	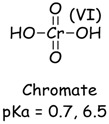

The solution pH has a pronounced effect on the Cr(VI) species and surface charge of TiO_2_. The pK_a1_ and pK_a2_ of chromic acid (shown in Table 1) are 0.7 and 6.5, respectively. The zero point of charge (ZPC) of P25 TiO_2_ is 6.8 [34]. Hence, the reduction of Cr(VI) was investigated as a function of solution pH. When the solution pH is less than 6.8, the TiO_2_ is positively charged and the predominant Cr(VI) species HCrO_4_^−^ and CrO_4_^2−^ are negatively charged. The photocatalytically initiated reactions mainly occur at or near the surface of TiO_2_ and thus electrostatic attraction between charged substrate and oppositely changed surface can lead to strong adsorption and enhance the reactivity or reduction of Cr(VI) at solution pH below 6.8. As the solution pH increases above 6.8, the TiO_2_ surface becomes progressively more negatively charged while the predominant Cr(VI) species is CrO_4_^2−^. The electrostatic repulsion between the negatively charged TiO_2_ surface and CrO_4_^2−^inhibits Cr(VI) adsorption and retards reduction. 

UV/TiO_2_ processes often following pseudo-first order kinetics according to Equation (6), below.

ln(C_0_/C) = kt (6)

where k is the rate constant of pseudo-first order model (min^−1^), t is time (min), C_0_ is the initial concentration of Cr(VI) and C is the concentration of Cr(VI) at the specific time.

**Figure 2:**
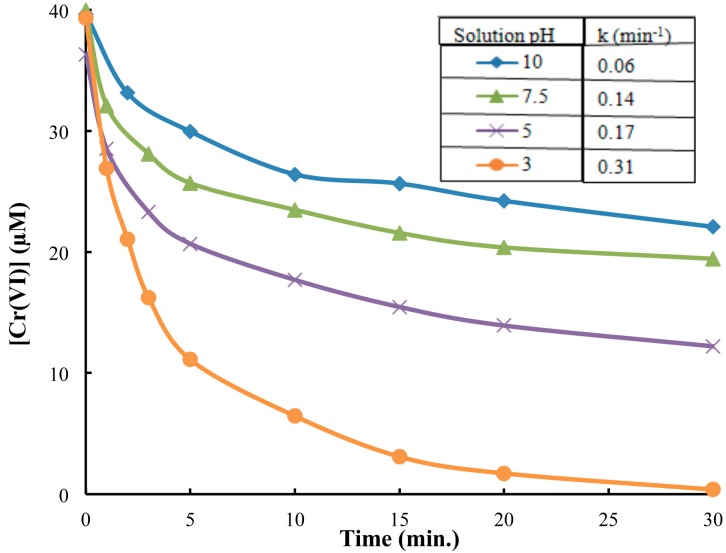
Investigation of reaction kinetics of Cr(VI) reduction as a function of solution pH. [Cr(VI)] = 40 µM; [TiO_2_] = 0.10 g/L, 350 nm.

The reduction of Cr(VI) was monitored as a function of irradiation time at several different initial Cr(VI) concentrations. The UV/TiO_2_ photocatalytic reduction of Cr(VI) was measured over a range of solution pH from ~3.0 to 10.0 with the fastest occurring under acidic conditions (Figure 2). The pH effect has been rationalized based on adsorption and the role of electrostatic interactions between TiO_2_ surface and chromate species which are attractive at low pH and repulsive at high pH [33].

### 2.2. The Reduction of Cr(VI) in Presence of As(V) at pH 3.0

**Figure 3:**
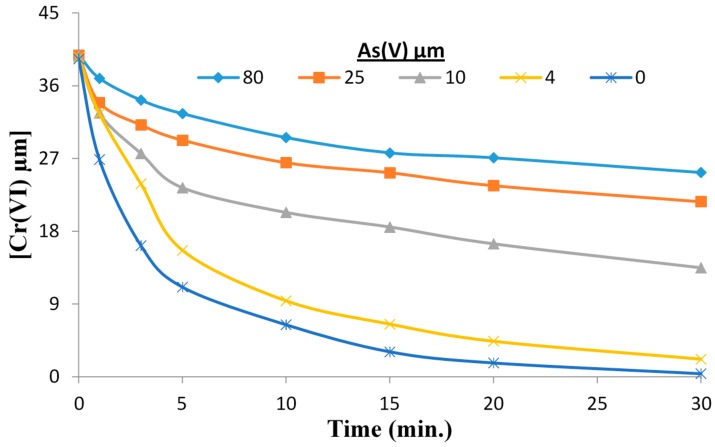
The reduction of Cr(VI) in the presence of As(V) at pH 3.0. [Cr(VI)] = 40 µM, [As(V)] = 0–80 µM, [TiO_2_] = 0.10 g/L, 350 nm.

**Figure 4:**
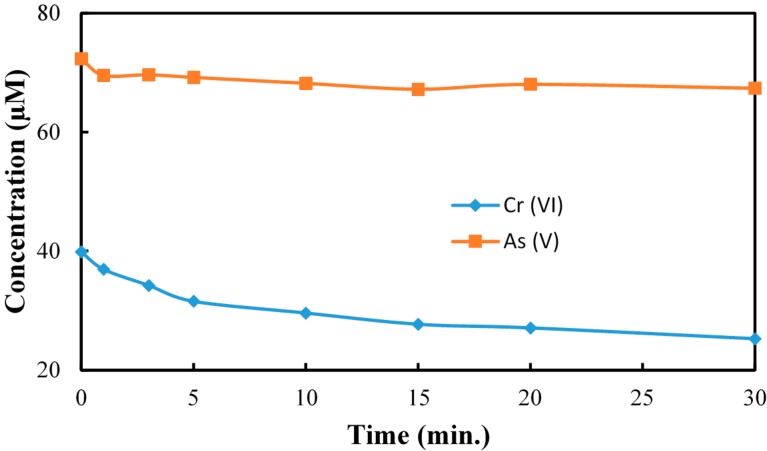
The reduction of As(V) in presence of Cr(VI). [Cr(VI)]_0_ = 40 µM, [As(V)]_0_ = 80 µM, pH = 3, [TiO_2_] = 0.10 g/L, 350 nm.

While transformation of Cr(VI) to Cr(III) can be achieved by employing UV and visible light- activated TiO_2_ materials, the effects of As and Cu on the reduction process have not been reported which is especially important for the treatment of CCA waste streams. The effect of As(V) presence on the reduction rate of Cr(VI) at pH 3.0 is illustrated in Figure 3. While significant reduction of Cr(VI) is observed, the rate of reduction decreased with increasing As(V) concentration. A number of literature reports describe the oxidation of As(III) to less toxic As(V) under TiO_2_ photocatalysis [34,35,36]. These reports also established TiO_2_ photocatalysis does not lead to the reduction of As(V) and thus under our experimental conditions As(V) did not compete with Cr(VI) for the conduction band electrons. We did not detect the presence of As(III) in the reaction solution following the TiO_2_ photocatalysis treatment. A modest inhibition of Cr(VI) reduction observed upon addition of As(V) and slight decrease of As(V) detected in solution relative to the initial concentration (Figure 4). These results are attributed to the competition between As(V) and Cr(VI) for adsorption at surface active sites where the conduction band electrons are available for reduction of the adsorbate.

### 2.3. The Reduction of Cr(VI) in Presence of Cu(II)

**Figure 5:**
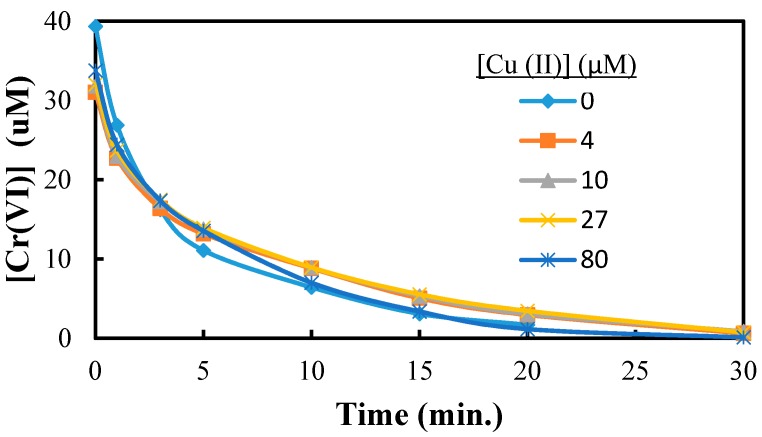
The reduction of Cr(VI) in the presence of Cu(II). [Cr(VI)] = 40 µM, [Cu (II)] = 0 µM–80 µM, pH = 3, [TiO_2_] = 0.10 g/L, 350 nm.

**Figure 6:**
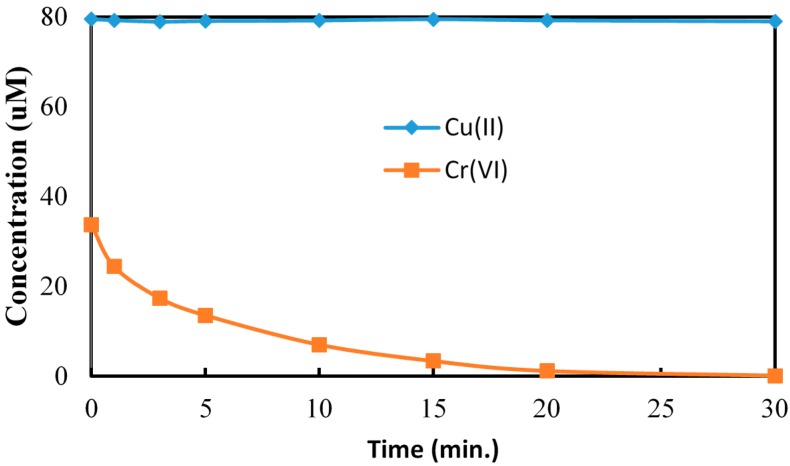
The reduction of Cu (II) in presence of Cr(VI). [Cr(VI)]_0_ = 40 µM, [Cu(II)]_0_ = 80 µM, pH = 3, [TiO_2_] = 0.10 g/L, 350 nm.

The effect of Cu(II), the third component in CCA on the reduction of Cr(VI) is shown in Figure 5. The addition of copper had no significant effect on the UV/TiO_2_ photocatalytic reduction of Cr(VI) over a range of Cu(II) concentrations. While Cu(II) ions have been used as conduction band electron scavengers during TiO_2_ photocatalysis, the presence of Cu(II) ions had no effect on the rate of reduction of Cr(VI) under our experimental conditions as illustrated in Figure 6. The concentration of Cu(II) was monitored using atomic absorption throughout the TiO_2_ photocatalytic reduction of Cr(VI). The changes in the concentration of Cu(II) as a function of irradiation time were insignificant, as shown in Figure 6, indicating no Cu(II) reduction occurred during our UV/TiO_2_ photocatalysis experiments [32]. These results demonstrate that Cu(II) as included in the CCA formulation does not compete with Cr(VI) for conduction band electrons or critical surface adsorption sites during TiO_2_ photocatalytic reduction of Cr(VI).

### 2.4. Cr(VI) Reduction in Presence of As(V) and Cu(II)

**Figure 7:**
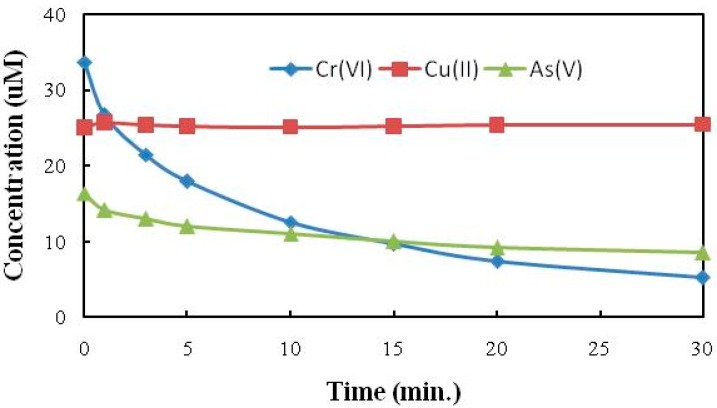
TiO_2_ photocatalysis of Cr(VI) in presence of As(V) and Cu(II). [Cr(VI)] = 40 µM, [As(V)] = 25 µM, [Cu(II)] = 25 µM, pH = 2, [TiO_2_] = 0.10 g/L, 350 nm.

**Figure 8:**
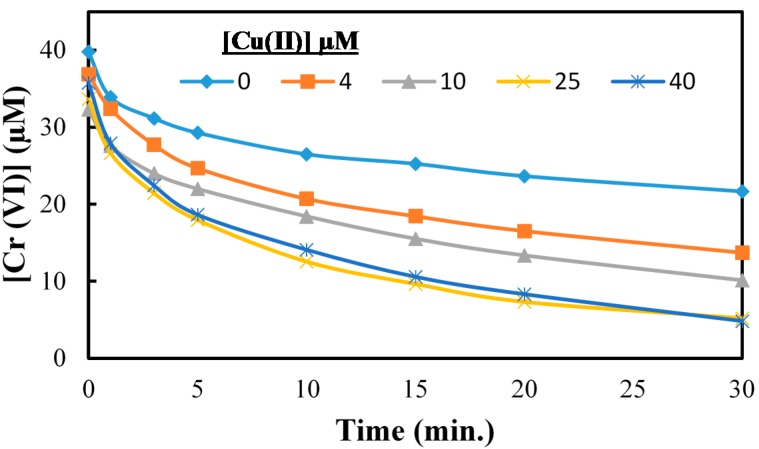
The effect of different Cu(II) concentration on Cr(VI) reduction in presence of As(V). [Cr(VI)] = 40 µM, [As(V)] = 25 µM, [Cu(II)] = 0–40 µM, pH = 3, [TiO_2_] = 0.10 g/L, 350 nm.

Treatment of CCA as a mixture is much more practical compared to the processes requiring separation of the metal ions and multiple treatment trains. With this in mind the cooperative effect of Cu(II) and As(V) on the TiO_2_ photocatalysis reduction of Cr(VI) to Cr(III) was investigated mimicking typical CCA formulations. TiO_2_ photocatalysis experiments were conducted in presence of 25 μM As(V), 25 μM Cu(II) and 40 μM Cr(VI) under 350 nm irradiation. The reaction suspension was collected and filtered at specific time intervals. Analysis of the solution as a function of treatment time showed that Cr(VI) is reduced to Cr(III) at only a slightly slower rate than the reduction of Cr(VI) alone, in the absence of As and Cu (Figure 7). While the initial concentration of Cu(II) remained unchanged throughout the photocatalytic treatment, the small decrease in the initial As(V) concentration relative to the starting concentration is assigned to strong adsorption on TiO_2_ surface. Although the addition of Cu(II) alone showed no effect on the rate of Cr(VI) reduction, in presence of 40 μm of Cr(VI) and 25 μm of As(V), addition of Cu(II) (≥25 μm) lead to a modest increase in the Cr(VI) reduction rate (Figure 8). The small enhancement may be due to interactions between As(V) and Cu(II) and/or competitive adsorption processes which reduces the adsorption of As for active sites on the surface of the TiO_2_ thus effectively improving the adsorption and subsequent reduction of Cr(VI).

**Figure 9:**
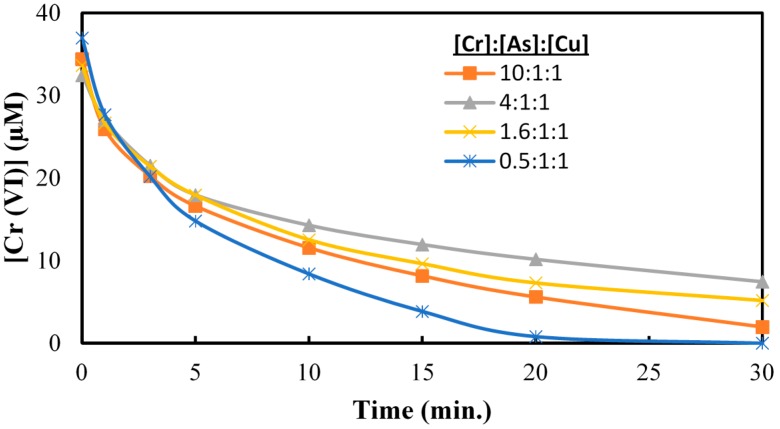
Cr(VI) reduction at different Cr(VI):As(V):Cu(II) ratios. pH = 3, [TiO_2_] = 0.10 g/L, 350 nm.

**Figure 10:**
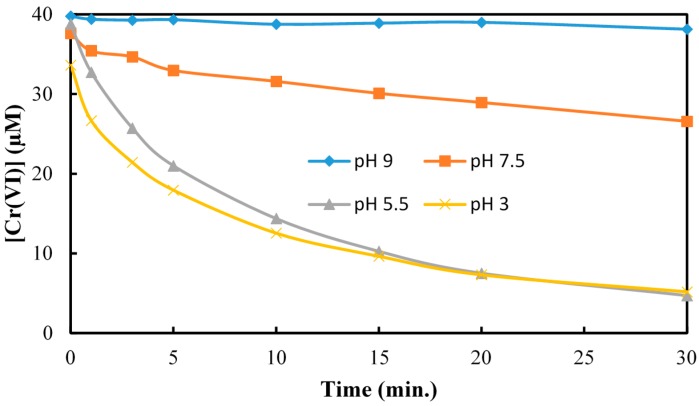
The pH effect on Cr(VI) reduction in presence of As (V) and Cu (II). Cr(VI):As(V):Cu(II) = 1.6:1:1. [TiO_2_] = 0.10 g/L, 350 nm.

The ratio of the initial concentrations of Cr(VI), As(V) and Cu(II) was varied during TiO_2_ photocatalysis to model common CCA formulations. At a 0.5:1:1 ratio of Cr(VI):As(V):Cu(II) the fastest and most extensive reduction of Cr(VI) was observed (Figure 9).These changes as a function of CCA formulation are attributed to competitive adsorption among the metals and not for the reactions with the conduction band electrons. The effect of pH on the reduction of 40 μm of Cr(VI) in presence of 25 μm of As(V) and 25 μm of Cu(II) is illustrated in Figure 10. This result suggests that the UV/TiO_2_ photocatalytic reduction of Cr(VI) is faster underacidic pH conditions both in the presence and absence of As(V) and Cu(II).

### 2.5. Effect of Hole Scavenger on Cr(VI) Reduction

Upon irradiation, TiO_2_ can absorb photons of ≤385 nm promoting an electron from the valence band to the conduction band forming an electron-hole pair. Rapid recombination of the electron and hole pair prevents redox transformation of adsorbates, and typically an electron scavenger is required to trap the electron and inhibit recombination. UV/TiO_2_ photocatalysis has been extensively used for oxidative transformation of pollutants and toxins with molecular oxygen purged through the system as an electron scavenger. Molecular oxygen acts as trap for the conduction band electron prolonging the lifetime of the hole and dramatically improving the probability to achieve oxidation of a surface adsorbed species. In the absence of a suitable electron trap little or no oxidative transformation is observed as photo-excitation energy is converted to thermal energy upon recombination.

For the case of Cr(VI), we chose to investigate the influence of adding an electron donor in an attempt to trap the hole, to inhibit recombination and extend the lifetime of the conduction band electron as the desired species to facilitate the reduction of Cr(VI). Formic acid can scavenge the valence band hole and is readily adsorbed onto the surface under neutral and acidic solution pH leading to an inhibition of recombination of electron and hole pairs on the TiO_2_ surface Equations (7)–(9).

(7) $$TiO_{2}\;\overset{hv}{⇌}\;h^{+}+\;e^{-}$$

(8) $$2h^{+}+\;2HCO_{2}^{-}\;\to 2CO_{2}+\;2H^{+}$$

(9) $$Cr\left(VI\right)+\;3e^{-}\;\to Cr(III)\;$$

**Figure 11:**
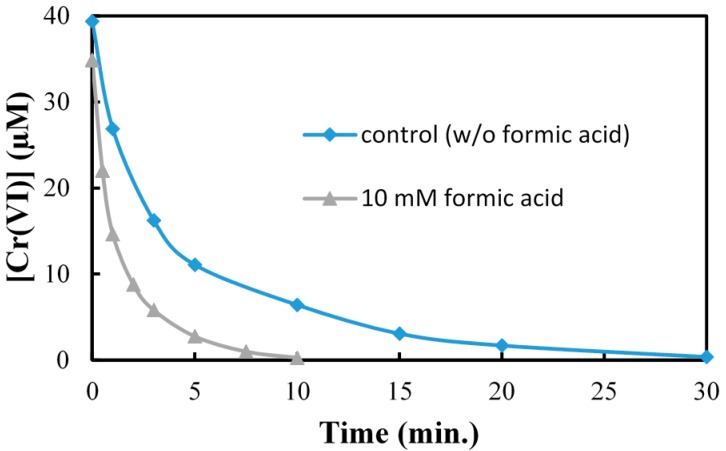
Effect of formic acid as hole scavenger on Cr(VI) reduction. [Cr (VI)] = 40 µM, [HCOOH] = 10 mM, [TiO_2_] = 0.10 g/L, 350 nm.

With this in mind formic acid was added to the reaction mixture prior to TiO_2_ photocatalytic treatment of Cr(VI).We observed a dramatic enhancement in the reduction rate of Cr(VI), indicating that formic can trap the hole to extend the lifetime of the electron enhancing the rate of Cr(VI) reduction (Figure 11). Since molecular oxygen does not affect the rate it does not appear reduction to superoxide anion radical and subsequent reaction with Cr(VI) change the observed rates of reduction.

## 3. Experimental

### 3.1. Materials

Degussa P25 TiO_2_ (CAS NO. 13463-67-7), a mixture of 80% anatase and 20% rutile with an average surface area of 50 m^2^/g, was donated by Degussa (Ridgefield, NJ, USA). Copper(II) nitrate trihydrate (99%–104%), sodium arsenate dibasic heptahydrate (99%) and sodium *meta*-arsenite (98%) were obtained from Sigma (St. Louis, MO, USA), while potassium chromate (99.8%), sodium hydroxide (99.4%), ammonium hydroxide (29.15%), trace metal grade nitric acid (67%–70%) and formic acid (88%) were purchased from Fisher (Waltham, MA, USA). All the chemicals were used without further purification. Millipore filtered water (18 MʶΩ cm) was obtained from a nanopure diamond lab water system (Barnstead Thermolyne Corporation, Dubuque, IA, USA) at room temperature. Volumetric glasswares were used to prepare all solutions.

### 3.2. Sample Preparation and Analysis

The concentration of TiO_2_ was 0.10 g/L, and the initial concentration of Cr(VI) was 40 µmol/L unless otherwise stated. As(III), As(V) and/or Cu(II) were added into TiO_2_ suspension based on experimental design. One hundred mL of TiO_2_ suspension was subject to an ultrasonic bath for 15 minutes to achieve a homogeneous suspension of the catalyst in the reaction vessel (Pyrex tube 12 × 1 in, 160 mL capacity, with a vented Teflon screw top). The suspension was magnetically stirred for 1.0 hour in the dark to allow equilibrium adsorption between target compound and TiO_2_. The solution pH was adjusted with 0.1 M HNO_3_ and 0.1 M NaOH and measured using a calibrated Pinnacle 530 pH meter (Corning, Vernon Hills, IL, USA).The solution was gently purged with the desired gas (air, oxygen or argon) for 15 min prior to and during irradiation. Irradiation of TiO_2_ suspension was conducted in a Rayonet photochemical reactor (model RPR-100, Southern New England Ultraviolet, Branford, Connecticut, USA), equipped with a cooling fan and 15 phosphor coated low-pressure mercury lamps (λ_max_ = 350 nm, light intensity = 5.2 ± 0.1 × 10^6^ photon/s/cm^3^). Five mL samples were taken at each specified time intervals, filtered through a 0.45 μm PTFE filter and subsequently analyzed.

The total copper and chromium were measured using an Analyst 600 atomic absorption spectrophotometer (Perkin–Elmer, Waltham, MA, USA) at 324.8 nm and 357.9 nm as recommended by the manufacturer. Determination of total arsenic was conducted using inductively coupled plasma mass spectroscopy (ICP-MS, HP 4500, Agilent, Santa Clara, CA, USA). The plasma and auxiliary gas flow rates of the ICP-MS were maintained at 15.4 and 1.0 L/min, respectively. Arsenic speciation of arsenate and arsenite and Cr(VI) were performed using a high-performance liquid chromatograph (HPLC) instrument equipped with a Lab Alliance series III digital pump (Fishersci, Waltham, MA, USA) and an ion exchange column coupled to ICP-MS detection. A PRP X-100 (250 mm × 4.6 mm, 10 μm particle size) anion-exchange column(Hamilton, North Quincy, MA, USA) was used to separate Cr(VI) and arsenic species with the mobile phase of 40 mM (pH 9.0) ammonium nitrate at the flow rate of 1.0 mL/min.

## 4. Conclusions

TiO_2_ photocatalysis is effective for the complete reduction of Cr(VI) to Cr(III) in the presence and absence of Cu(II) and As(V). The reduction process is effective over a range of solution pH and under oxygen, air, or argon saturated conditions. The pH effects indicate that the reduction is favorable under acidic solution conditions due to enhanced adsorption through electrostatic attraction between positively charged TiO_2_ surface and anionic chromate species. The addition of As(V) in the reaction mixture slightly reduces the reduction rate of Cr(VI) which is assigned to competitive adsorption between As(V) and Cr(VI) and competition for reduction processes since As(V) is not reduced under the experimental conditions. Although Cu(II) can be reduced during TiO_2_ photocatalysis, we observe no reduction of Cu(II) and a slight enhancement in Cr(VI) reduction under very specific conditions. The effective reduction of Cr(VI) is achieved in the presence of As(V) and Cu(II) at different ratios. The addition of formic acid to the reaction solution shows a significant acceleration in the reduction of Cr(VI). Our study clearly demonstrates UV/TiO_2_ photocatalysis is a promising single treatment process for the remediation of Cr(VI) mixed wastes under a variety of conditions.

## References

[1] Cooper P.A. (1994). Leaching of CCA: Is it a problem?. Environmental Considerations in the Manufacturing, Use and Disposal of Preservative-Treated Wood.

[2] Joanna S.W., KongHwa C. (2008). Extraction of chromated copper arsenate from wood wastes using green solvent supercritical carbon dioxide. J. Hazard. Mater..

[3] Gordon T., Spanier J., Butala J.H., Li P., Rossman T.G. (2002). *In vitro* bioavailability of heavy metals in pressure treated wood dust. J. Toxicol. Sci..

[4] Stillwell D., Gorny K. (1997). Contamination of soil with copper, chromium, and arsenic under decks built from pressure treated wood. Bull. Environ. Contam. Toxicol..

[5] WHO (2003). Arsenic in Drinking-Water, Background Document for Development of WHO Guidelines for Drinking-Water Quality.

[6] WHO (2003). Chromium in Drinking-Water, Background Document for Development of WHO Guidelines for Drinking-Water Quality.

[7] ATSDR (2004). Toxicological Profile for Copper, Background and Environmental Exposures to Copper in the United States.

[8] Mason R.W., Edwards I.R. (1989). Acute toxicity of combinations of sodium dichromate, sodium arsenate and copper sulphate in the rat. Comp. Biochem. Physiol. Part C.

[9] Humphrey D.G. (2002). The chemistry of chromated copper arsenate wood preservatives. Rev. Inorg. Chem..

[10] Cooper P.A. A review of issues and technical options for managing spent CCA treated wood. Proceedings of the AWPA Annual Meeting.

[11] El-Fatah S.M., Goto M., Kodama A., Hirose T. (2004). Supercritical fluid extraction of hazardous metals from CCA wood. J. Supercrit. Fluids.

[12] Gyu-Hyeok K., Jong-Bum R., li-Gon K., Yun-Sang S. (2004). Optimization of hydrogen peroxide extraction conditions for CCA removal from treated wood by response surface methodology. For. Prod. J..

[13] Kartal S.N. (2003). Removal of copper, chromium, and arsenic from CCA-C treated wood by EDTA extraction. Waste Manag..

[14] Clausen C.A., Smith R.L. (1998). Removal of CCA from treated wood by oxalic acid extraction, steam explosion, and bacterial fermentation. J. Ind. Microbiol. Biotechnol..

[15] Janin A., Blais J.F., Mercier G., Drogui P. (2009). Optimization of a chemical leaching process for decontamination of CCA-treated wood. J. Hazard. Mater..

[16] Khan B.I., Solo-Gabriele H.M., Townsend T.G., Cai Y. (2006). Release of arsenic to the environment from CCA-treated wood.1.Leaching and speciation during service. Environ. Sci. Technol..

[17] Townsens T., Solo-Gabriele H., Tolaymat T., Stook K., Hosein N. (2003). Chromium, Copper, and Arsenic Concentrations in Soil Underneath CCA-Treated Wood Structures. Soil Sediment Contam..

[18] Janin A., Zaviska F., Drogui P., Blais J., Mercier G. (2009). Selective recovery of metals in leachate from chromated copper arsenate treated wastes using electrochemical technology and chemical precipitation. Hydrometallurgy.

[19] Nygren O., Nilsson C.A. (1993). Determination and speciation of chromium, copper and arsenic in wood and dust from CCA-impregnated timber. Analusis.

[20] Jiang W., Cai Q., Xu W., Yang M., Cai Y., Dionysiou D.D., O’Shea K.E. (2014). Cr(VI) Adsorption and Reduction by Humic Acid Coatedon Magnetite. Environ. Sci. Technol..

[21] Gupta V.K., Agarwal S., Saleh T.A. (2011). Chromium removal by combining the magnetic properties of iron oxide with adsorption properties of carbon nanotubes. Water Res..

[22] Kongsricharoern N., Polprasert C. (1996). Chromium removal by a bipolar electro-chemical precipitation process. Water Sci. Technol..

[23] Aarthi T., Madras G. (2008). Photocatalytic reduction of metals in presence of combustion synthesized nano-TiO_2_. Catal. Commun..

[24] Chenthamarakshan C.R., Rajeshwar K. (2000). Heterogeneous Photocatalytic Reduction of Cr(VI) in UV-Irradiated Titania Suspensions: Effect of Protons, Ammonium Ionsand, Other Interfacial Aspects. Langmuir.

[25] Lin W.Y., Wei C., Rajeshwar K. (1993). Photocatalytic reduction and immobilization of hexavalent chromium at titanium dioxide in aqueous basic media. J. Electrochem. Soc..

[26] Parida K., Mishra K.G., Dash S.K. (2012). Adsorption of toxic metal ion Cr(VI) from aqueous state by TiO_2_-MCM-41: Equilibrium and kinetic studies. J. Hazard. Mater..

[27] Zhang L., Zhang Y. (2006). Adsorption characteristics of hexavalent chromium on HCB/TiO2. Appl. Surf. Sci..

[28] Zhang Y.C., Yang M., Zhang G., Dionysiou D.D. (2013). HNO_3_-involved one-step low temperature solvothermal synthesis of N-doped TiO_2_ nanocrystals for efficient photocatalytic reduction of Cr(VI) in water. Appl. Catal. B.

[29] Yoon J., Shim E., Joo H. (2009). Photocatalytic reduction of hexavalent chromium (Cr(VI)) using rotating TiO_2_ mesh. Korean J. Chem. Eng..

[30] Wang Q., Shang Q., Zhu T., Zhao F. (2011). Efficient photoelectrocatalytic reduction of Cr(VI) usingTiO_2_ nanotube arrays as the photoanode and a large-area titanium mesh as the photocathode. J. Mol. Catal. A.

[31] Xu X.R., Li H.B., Gu J.D. (2006). Simultaneous decontamination of hexavalent chromium and methyl *tert*-butyl ether by UV/TiO_2_ process. Chemosphere.

[32] Liu W., Ni J., Yin X. (2014). Synergy of photocatalysis and adsorption for simultaneous removal of Cr(VI) and Cr(III) withTiO_2_ and titanate nanotubes. Water Res..

[33] Prairie M.R., Evans L.R., Stange B., Martinez S.L. (1993). An investigation of titanium dioxide photocatalysis for the treatment of water contaminated with metals and organic chemicals. Environ. Sci. Technol..

[34] Xu T., Cai Y., O’Shea K.E. (2007). Adsorption and Photocatalyzed Oxidation of Methylated Arsenic Species in TiO_2_Suspensions. Environ. Sci. Technol..

[35] Zheng S., Cai Y., O’Shea K.E. (2010). TiO_2_photocatalytic degradation of phenylarsonic acid. J. Photochem. Photobiol. A.

[36] Zheng S., Jiang W., Cai Y., Dionysiou D.D., O’Shea K.E. (2014). Adsorption and photocatalytic degradation of aromatic organoarsenic compounds in TiO_2_suspension. Catal. Today.

